# Should the Dermatological Assessment of Patients with Inflammatory Bowel Disease Become Standard during Qualifications for Biological Treatment? A Retrospective, Single-Center Experience from a Tertiary Center

**DOI:** 10.3390/jcm13175213

**Published:** 2024-09-02

**Authors:** Konrad Lewandowski, Magdalena Kaniewska, Edyta Tulewicz-Marti, Martyna Głuszek-Osuch, Piotr Ciechanowicz, Irena Walecka, Grażyna Rydzewska

**Affiliations:** 1 Clinical Department of Internal Medicine and Gastroenterology, National Medical Institute of the Ministry of the Interior and Administration, 02-507 Warsaw, Poland; dr.k.lewandowski@icloud.com (K.L.); e.tulewicz@gmail.com (E.T.-M.); martynaosuch1@gmail.com (M.G.-O.); grazyna.rydzewska@cskmswia.gov.pl (G.R.); 2 Collegium Medicum, Jan Kochanowski University, 25-369 Kielce, Poland; 3 Department of Dermatology, Centre of Postgraduate Medical Education, 01-813 Warsaw, Poland; piotr.ciechanowicz91@gmail.com (P.C.); irena.walecka@cskmswia.gov.pl (I.W.); 4 Department of Dermatology, National Institute of Medicine of the Ministry of Interior and Administration, 02-507 Warsaw, Poland

**Keywords:** biological treatment, dermal lesions, extraintestinal manifestations, inflammatory bowel disease, cutaneous malignancies

## Abstract

**Background**: Oncological anxiety associated with biological therapy is a particular challenge in inflammatory bowel disease (IBD), and it has raised questions about the need for the dermatological assessment of the skin before starting biological therapy. **Methods**: The aim of this study was to assess the frequency of dermal lesions, including cutaneous malignancies, in IBD patients. This retrospective, single-center study evaluated 805 IBD patients who qualified for biological treatment and were subjected to a dermatological assessment. **Results**: Dermal lesions (DLs) were found in 15.5% (125) of IBD patients. A risk factor for DLs was higher with body mass index (OR = 1.08, 95% CI [1.02; 1.14], *p* = 0.007). Surprisingly, there was no effect of thiopurines between the groups with and without DLs (90.4% vs. 84.6%, MD = 0.06, 95% CI [0.01; 0.12], *p* = 0.118). Moreover, cutaneous malignancies were diagnosed in 9 cases (1.1%), including 4 basal cell carcinomas, 4 squamous cell carcinomas, and 1 melanoma skin cancer. Only 13.4% of patients complied with our strict policy of skin surveillance every 6–8 months. **Conclusions:** DLs, including cutaneous malignancies, are common in patients with IBD, making skin monitoring at the initiation of biological treatment an extremely useful tool. The lack of effect of the drugs used suggests that skin surveillance is necessary in all IBD patients. The low compliance of skin monitoring among immunosuppressed patients indicates the need for better education on the prevention of cutaneous malignancies.

## 1. Introduction

Inflammatory bowel disease (IBD) includes ulcerative colitis and Crohn’s disease, which are incurable autoimmune diseases with periods of flare-ups and remission [1,2,3,4]. Since many pro-inflammatory pathways are stimulated during exacerbations, treatments that modulate the immune system are used: immunosuppressive drugs (thiopurines and methotrexate), biologics, and small molecules. Their prolonged intervention in suppressing the immune system may increase the oncological risk in patients with IBD [5]. Many societies recommend surveillance related to the suppression of the immune system before and during biological treatment, and this is especially true for various types of infections [1,2,3,4,5]. However, there are no clear recommendations for cancer screening other than those specific to complications of IBD, such as colorectal cancer or biliary tract cancer, in patients with IBD and primary sclerosing cholangitis (PSC) [5,6,7].

One of the organs at risk of malignancy is the skin. Various types of dermal lesions are quite common in patients with IBD, with estimates ranging from 16.4% to 30.9%, making the diagnosis of these malignancies much more difficult [8]. The occurrence of a cutaneous malignancy is often associated with the drugs administered. Associations have been shown between thiopurines and non-melanoma skin cancers (NMSCs) and between the anti-tumor necrosis factor (TNF) and melanoma skin cancers (MSCs). In addition, the presence of certain genes may increase the likelihood of developing cutaneous malignancies [9]. Despite this, there is still no adequate algorithm in place to regulate surveillance at appropriate intervals, which makes the management of these patients much more difficult [9,10].

Immune homeostasis is a common phenomenon in the gastrointestinal tract and skin, with both systems being exposed to environmental pathogens. In gastroenterology, biological therapy is currently the fastest-growing treatment, which is probably related to increasing financial investment in the treatment of these patients. This calls for more emphasis on patient verification before and during biological therapy in order to prevent the development of skin complications, with a particular focus on cutaneous malignancies. The self-reported question of the role of dermatologists in the qualification of patients for biological treatment as a routine procedure was the basis of this analysis.

Dermal lesions, including cutaneous malignancies in patients with IBD, are common, and their occurrence may reduce the safety of or limit biological therapy. Therefore, we conducted this study to assess the prevalence of cutaneous malignancies and the usefulness of skin surveillance in IBD patients scheduled for biological therapy.

## 2. Materials and Methods

### 2.1. Study Design and Patients

This was a single-center, retrospective study conducted at the Department of Gastroenterology and Internal Medicine and the Department of Dermatology of the National Medical Institute of the Ministry of the Interior and Administration in Warsaw, Poland, between January 2016 and April 2023. We included a total of 805 IBD patients in the study, who were at least 18 years of age and with a histologically confirmed diagnosis of IBD according to the criteria of the Polish Society of Gastroenterology and the European Crohn’s and Colitis Organisation (ECCO) [1,2,3,4]. For IBD patients on immunosuppressive therapy (thiopurines) at our center, a restrictive skin assessment strategy was carried out every 6–8 months or urgently if skin lesions occurred [10,11,12]. Other inclusion criterion were a negative history for biological therapy and a skin assessment carried out by an experienced dermatologist. There were no exclusion criteria.

### 2.2. Outcomes

The primary outcome was to estimate the prevalence of dermal lesions, including cutaneous malignancies. The secondary outcomes were an estimate of risk factors for dermal lesions and an assessment of the usefulness of dermatological skin examinations when qualifying for biological treatment.

### 2.3. Statistical Analysis

Statistical analysis was run for the 805 biologically naïve IBD patients. Categorical variables are described with the number of observations and the frequency (%). Numerical variables are described with means and standard deviation or medians and the 1st and 3rd quartiles, depending on normality. Normality was tested with a Shapiro–Wilk test. If its outcome indicated normality (*p* > 0.05), the distribution was treated as normal; otherwise, if skewness was between −1 and 1 and kurtosis was between 2 and 4, the distribution was also treated as normal. In other instances, the distribution was treated as non-normal. The homogeneity of variances was tested with Levene’s test. The differences between groups for continuous parameters were assessed using Student’s independent *t*-test, Welch’s independent *t*-test, or the Mann–Whitney U test, as appropriate. The differences between groups for categorical variables were assessed with Pearson’s Chi-square test or Fisher’s exact test, as appropriate, and described with Cramer’s V. The mean/median difference and Cramer’s V are presented with 95% confidence intervals. A logistic two-step regression approach was used to identify significant predictors of dermal lesions (with univariable models for each variable in the first step and multivariable models in the second step). The selection of variables for multivariable models was based on the *p*-value determined in the first step, under the additional condition of the *p*-value being no higher than 0.250, and further using the stepwise selection procedure. Multivariable models were assessed with Nagelkerke’s R2, Hosmer and Lemeshow’s test, and VIF. The predictors’ effect was described with a logistic regression coefficient, standard error, and odds ratio (OR), along with 95% confidence intervals. All statistical calculations assumed an alpha value of 0.05. The analysis was run using the software program R, version 4.1.2. The statistical analysis was conducted using IBM SPSS, version 25.0.0.2 (IBM Corp., Armonk, New York, NY, USA).

## 3. Results

The total group of patients with IBD who were referred for biological treatment consisted of 805 patients, out of which 43.2% were female. The average age was 36.84 ± 12.49 years. Dermal lesions were found in 15.5% (125) of patients at the time of qualification for biological treatment, of which 9 (1.1%) were cutaneous malignancies: 4 basal cell carcinomas (BCCs), 4 squamous cell carcinomas (SCCs), and 1 melanoma skin cancer (MSC). A detailed description of the study parameters for the group is presented in Table 1.

Both groups (with and without dermal lesions) were compared. Significant differences were found in body mass index (BMI), which was higher in patients with dermal lesions (MD = 1.23, 95% CI [0.32; 1.65], *p* = 0.004). Steroid resistance significantly differentiated the patients with dermal lesions from those without DLs. The former group included a lower proportion of patients with steroid dependence (84.6% vs. 92.9%) than the group without dermal lesions and a higher proportion of patients with steroid resistance (13.6% vs. 7.1%; V = 0.09, 95% CI [0.01; 0.17], *p* = 0.022) (Table 2). For this group, male sex and a higher BMI increased the odds of dermal lesions. The men had 49% higher odds of developing DLs than the women (OR = 1.49, 95% CI [1.01; 2.23], *p* = 0.049). A BMI of 1 kg/m^2^ higher resulted in 8% higher odds (OR = 1.08, 95% CI [1.02; 1.14], *p* = 0.005). Steroid dependence was associated with 52% lower odds compared to steroid resistance (OR = 0.48, 95% CI [0.27; 0.89], *p* = 0.015). Statistical significance was not confirmed for other predictor variables, including 5-aminosalicylic acid (5-ASA), steroids, thiopurines, and the type of IBD (*p* > 0.05).

The multivariable regression step identified that a BMI of 1 kg/m^2^ produced higher results in 8% higher odds of DLs (OR = 1.08, 95% CI [1.02; 1.14], *p* = 0.007). Steroid dependence was associated with 55% lower odds compared to those resistant to steroids (OR = 0.45, 95% CI [0.25; 0.83], *p* = 0.008). The other predictors analyzed in the multivariable regression did not have a significant impact on the risk when considered together (due to the multivariable model step) (Table 3).

Hosmer and Lemeshow’s test resulted in a *p*-value of 0.497, indicating a good model fit. The fit of the model was additionally assessed with Nagelkerke’s R2, which was found to be 4.5%, indicating the model’s restricted ability to predict the complete odds of dermal lesions in a patient. The independent variables’ collinearity was assessed with VIF, which ranged from 1.01 to 1.02 and indicated no collinearity.

## 4. Discussion

**Figure 1:**
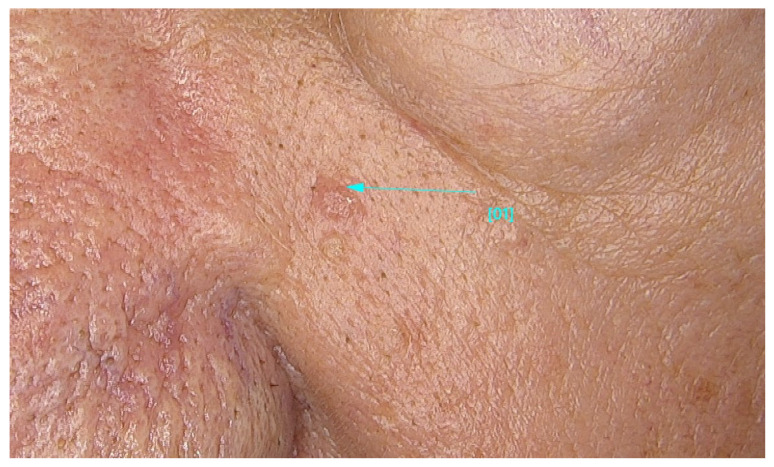
Basal cell carcinoma (BCC).

**Figure 2:**
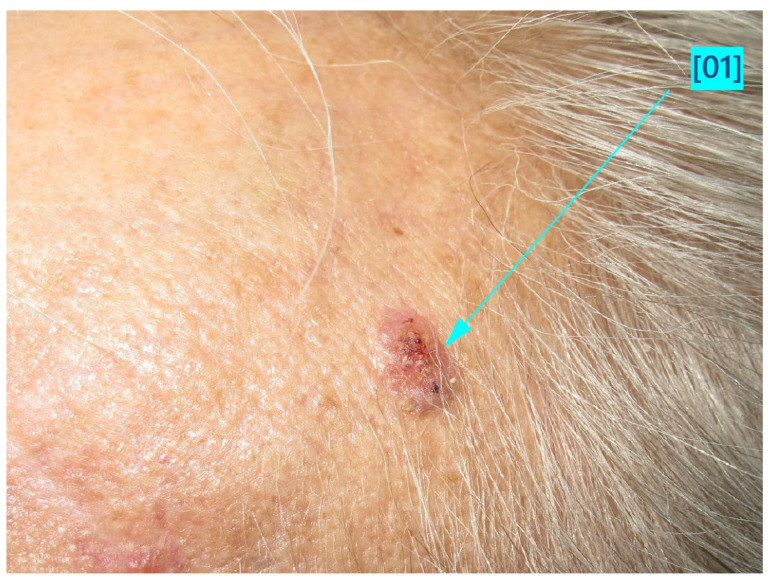
Squamous cell carcinoma (SCC).

**Figure 3:**
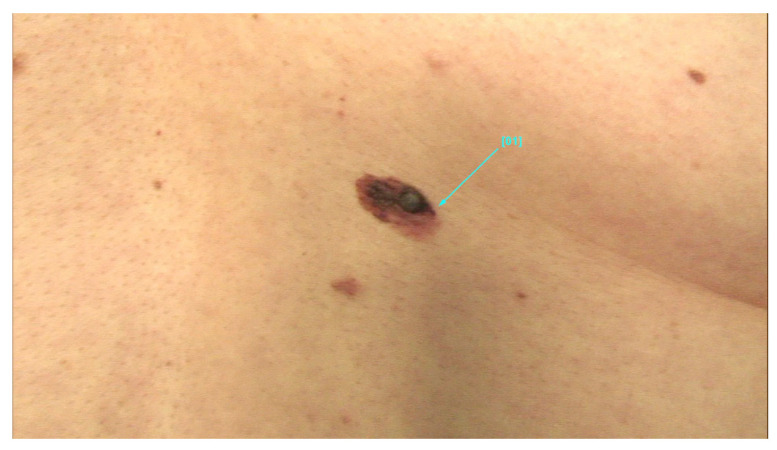
Melanoma skin cancer (MSC).

**Figure 4:**
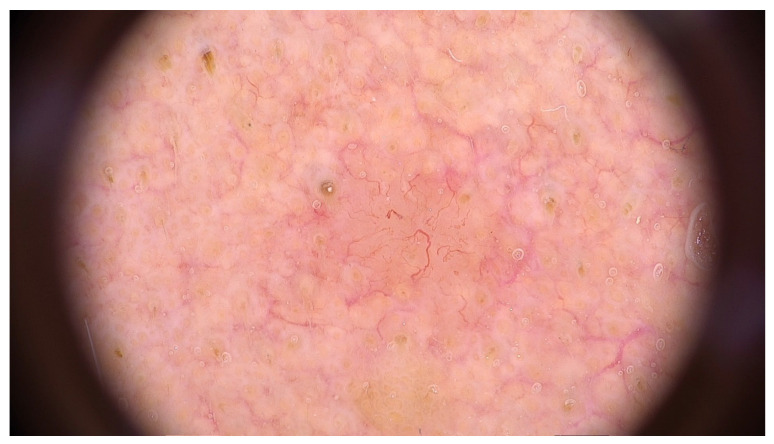
Videodermoscopy of basal cell carcinoma.

**Figure 5:**
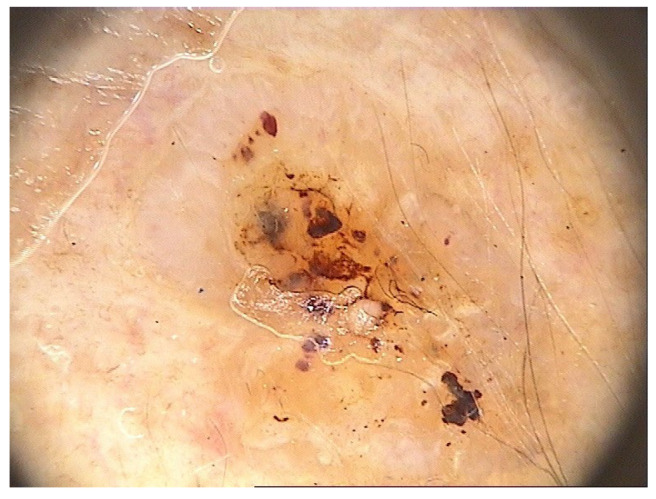
Videodermoscopy of squamous cell carcinoma.

**Figure 6:**
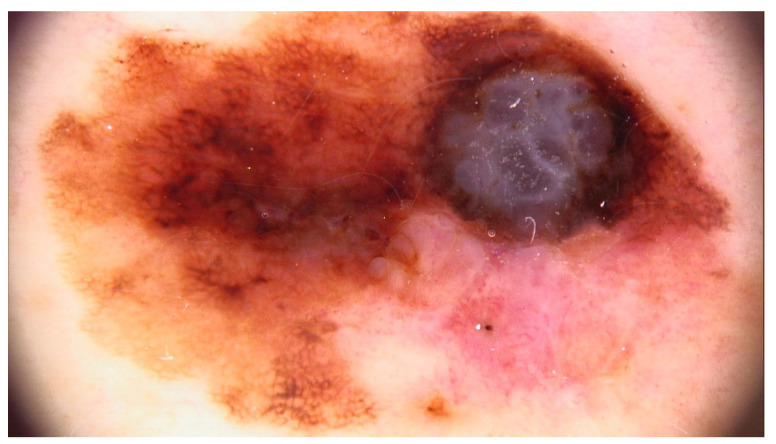
Videodermoscopy of melanoma skin cancer.

The main findings from our study were the presence of dermal lesions among 15.5% of (125) patients with nine (1.1%) cutaneous malignancies: 4 BCCs, 4 SCCs, and 1 MSC. Moreover, only 13.4% (92) of the 688 patients followed our recommendation of restrictive skin surveillance every 6–8 months. These cutaneous malignancies are shown in Figure 1, Figure 2 and Figure 3, and the videodermoscopy images of these lesions are presented in Figure 4, Figure 5 and Figure 6. 

Many scientific societies recommend skin surveillance in patients treated with thiopurines or TNF, but there is no precise algorithm for determining the interval between skin examinations [1,2,3,4]. In our center, following the recommendations from transplantology, strict supervision is performed every 6–8 months, the primary goal of which is to find changes at an early stage [13,14,15]. However, our results did not confirm the influence of thiopurines on the development of dermal lesions, which suggests the need for surveillance in all IBD patients. The number of patients diagnosed with cutaneous malignancies was too low to assess the relationship between their presence and thiopurines.

In 2019, Huang et al. published an analysis of 13 studies involving 149,198 patients. It suggested that thiopurine use in IBD significantly increases the risk of NMSC (RR = 1.88, 95% CI [1.48; 2.38]). In our study, among the 805 patients with IBD, 8 patients (0.99%) with NMSC were found, each of whom was treated with thiopurines, which could have influenced the development of cutaneous malignancies. In their conclusion, the authors emphasized the need for daily skin protection and routine skin screening in patients with a history of exposure to thiopurines [16]. They do not indicate a specific time interval between skin assessments, which remains crucial in terms of issuing precise recommendations for patients. In our study, MSC was found in only one patient who had received thiopurine, but Huang et al. did not report that the association between thiopurine use and MSC was statistically significant in any of the cohort studies (fixed effects: RR = 1.30, 95% CI [0.57; 2.99], *p* = 0.531, Q = 1.18, *p* for heterogeneity = 0.277, and I2 = 15.5%) or nested controls (random effects: RR = 1.20, 95% CI [0.87; 1.67], *p* = 0.266, Q = 0.44, *p* for heterogeneity = 0.505, and I2 < 0.1%). When pooled, the relative risk was 1.22 (95% CI [0.90; 1.65], *p* = 0.206), with no significant inter-study heterogeneity (I2 < 0.1%, *p* = 0.646) [16].

A finding of dermal lesions in a patient with IBD often raises many unknowns; in our group, lesions were found in as many as 15.5% (125) of patients. In addition to cancer lesions, the following types were found: xerosis and eczema in 44 (5.5%) cases, erythema nodosum in 21 (2.6%) cases, pyoderma gangrenosum in 17 (2.1%) cases, hidradenitis suppurativa in 15 (1.9%) cases, urticaria in 11 (1.4%) cases, psoriasis in 9 (1.1%) cases, vitiligo in 5 (0.6%) cases, erythema multiforme in 2 (0.2%) cases, phlebitis in 1 (0.1%) case, and alopecia areata/totalis and/or vitiligo in 2 (0.2%) cases. Although the presence of extra-intestinal manifestations (EIMs) ranges from 5 to 50%, the skin is often involved, with dermal lesions occurring in up to 15% of patients; we recorded a similar frequency [17,18,19]. In the multivariable regression model, a BMI higher than 1 kg/m^2^ was identified as a risk factor, resulting in 8% higher odds (OR = 1.08, 95% CI [1.02; 1.14], *p* = 0.007). In several studies, this relationship was associated primarily with an increase in the amount of pro-inflammatory cytokines, which may cause more frequent skin lesions in patients with a higher BMI [20,21,22].

However, the goal of optimal care for a patient with IBD should not only be to treat IBD or diagnose and appropriately treat EIM but should also involve long-term prevention to maintain health [1,2]. In terms of prophylaxis, apart from preventing infections, it is also crucial to prevent cancers because they are significantly more common in the IBD population [1,2,3,4,19]. The prevention and surveillance of various cancers are also the responsibility of IBD physicians. Unfortunately, there are no accurate data on the prevalence of cutaneous malignancies in the IBD population. There are, however, data on the US population, which clearly cannot be extrapolated to all IBD patients but which do raise enough concern to warrant rigorous surveillance of the skin. MSC rates are increasing year on year, with the American Cancer Society estimating that there were 91,270 new cases and 9320 deaths in 2018. NMSC is the most common of all types of cancer, with approximately 3.3 million Americans being diagnosed with it each year. BCC and SCC represent 80% and 20% of cases, respectively. Nevertheless, death from these cancers is not common. About 2000 people die of NMSC each year in the USA [23]. Therefore, it should be assumed that the incidence of cutaneous malignancies will increase, not only in the general population but also in IBD. To date, no randomized controlled trials have proven the effectiveness of skin surveillance, but observational studies suggest a reduction in the risk of death due to the detection of less advanced forms of cutaneous malignancies [13,24,25,26,27].

The key goal in prevention therapy for cutaneous malignancies should be to determine the interval between skin examinations. Such recommendations have been issued for patients after organ transplantation who are on dual immunosuppressive therapy, which is not unknown in IBD therapy [1,2]. Several dermatological scientific societies recommend a strict skin surveillance policy for patients on immunosuppressive therapy, during which a dermatologist evaluates the skin by dermoscopy every 6–8 months. Such management, despite the lack of randomized control trials, has contributed to the greater detection of NMSC and MSC at an early stage and reduced the overall risk of death compared to the general population [11,12]. The practice of checking the skin every 6–8 months may also become a potential threat to the healthcare system, not only due to the aging nature of the population and the constantly increasing frequency of both diseases but also due to the significant overload of dermatologists [13,28]. An interesting solution may be teledermatology, which increases access to screening tests for cutaneous malignancies. In 2017, a systematic review was carried out, confirming the usefulness of teledermatology for shortening wait times and improving patient satisfaction, but this method is not without its drawbacks [29]. Although a clear advantage of dermatological examination over teledermatology has been found, it is considered a supplementary screening option before an in-person visit to a dermatologist [30]. The lack of unequivocal evidence in the form of research or recommendations, apart from encouraging scientists to conduct randomized trials on this topic, should encourage doctors to increase patients’ self-awareness. Routine skin self-examinations (SSEs) have been shown to increase the early detection of cutaneous malignancies. A 2010 review of 15 studies found SSE to have low sensitivity (25–93%) and high specificity (83–97%). Nevertheless, it has also been found that educational interventions can improve the diagnostic accuracy of SSE as well as the patient’s ability to make appropriate decisions about the need for professional care [31]. In addition to SSEs, physicians should also advise patients to avoid tanning beds and to apply ultraviolet protection to the skin [7,27]. Similar conclusions were reached by a group of researchers from Italy, who compared the effect of developing a cutaneous malignancy through phototherapy and TNF biologic treatment in patients with psoriasis. The authors emphasized the need for routine dermatological assessment before starting biologics and for increased vigilance in cutaneous malignancy surveillance during treatment, especially in patients at risk (older age, a history of previous cutaneous malignancies, a family history of cutaneous malignancies and previous immunosuppressive therapy) [32].

An undoubted strength of this study is the presentation of the beneficial effect of skin supervision in patients during qualification for biological treatment and the incidence of dermal lesions, including cutaneous malignancies in patients with IBD. At the same time, this study had several limitations: its retrospective nature and the lack of a control group. We did not evaluate the association of a cumulative dose of thiopurine; thus, the lack of effect for developing dermal lesions could potentially weaken the results of our study. Furthermore, it should also be highlighted that there was no analysis of the cost-effectiveness of dermatological assessments in IBD patients.

## 5. Conclusions

We observed that dermal lesions are common in patients with IBD. More than 1% of patients had cutaneous malignancies, making skin surveillance when initiating biological therapy an extremely useful tool that can detect lesions at an early stage while increasing patient safety during biological treatment. The frequent occurrence of dermal lesions (including cutaneous malignancy), irrespective of previous medication use, indicates the need for skin surveillance in all IBD patients. In contrast, the low compliance of skin monitoring in immunosuppressed patients indicates the need for better education for the prevention of cutaneous malignancies.

## Figures and Tables

**Table 1:** Characteristics of patients with IBD who were qualified for biological treatment.

Variable	*n* = 805
Age, years, mean ± SD	36.84 ± 12.49
Gender	
Female	348 (43.2)
Male	457 (56.8)
BMI, kg/m^2^, mean ± SD	23.73 ± 3.51
IBD	
Ulcerative colitis	196 (24.3)
Crohn’s disease	609 (75.7)
Crohn’s disease, location	
Small intestine	76 (9.4)
Large intestine	48 (6.0)
Ileocecal	510 (63.4)
Ulcerative colitis, location	
Pancolitis	183 (22.7)
Left-sided	13 (1.6)
Proctitis	22 (2.7)
Steroids	
Dependent	740 (91.9)
Resistant	65 (8.1)
5-ASA	244 (30.3)
Steroids	499 (61.9)
Thiopurine	688 (85.5)
Dermal lesions, any	125 (15.5)
Erythema nodosum	21 (2.6)
Pyoderma gangrenosum	17 (2.1)
Psoriasis	9 (1.1)
Hidradenitis suppurativa	15 (1.9)
Vitiligo	5 (0.6)
Phlebitis	1 (0.1)
Erythema multiforme	2 (0.2)
Urticaria	11 (1.4)
Xerosis and eczema	44 (5.5)
Cutaneous infections	0 (0.0)
Psorasiform reactions	0 (0.0)
Cutaneous malignancies	9 (1.1)
Lupus-like symptoms	0 (0.0)
Vasculitis	0 (0.0)
Alopecia areata/totalis and/or vitiligo	2 (0.2)
Regular dermatological visits	92 (13.4) *

5-ASA—5-aminosalicylic acid, SD—standard deviation, IBD—inflammatory bowel disease, and IQR—interquartile range; * calculation only for the 688 patients on thiopurines.

**Table 2:** Comparison of patients with and without dermal lesions.

Variable	Patients with Dermal Lesions (*n* = 125)	Patients without Dermal Lesions (*n* = 680)	MD/Cramer’s V (95% CI)	*p*-Value
Age, years, mean ± SD	37.45 ± 12.03	36.73 ± 12.57	0.72 (−1.67; 3.10)	0.555
Gender				
Female	44 (35.2)	304 (44.7)	0.07 (0.01; 0.13)	0.061
Male	81 (64.8)	376 (55.3)		
BMI, kg/m^2^, median (IQR)	24.81 (22.13; 26.57)	23.58 (20.81; 26.03)	1.23 (0.32; 1.65)	**0.004**
IBD				
Ulcerative colitis	29 (23.2)	167 (24.6)	0.01 (0.00; 0.08)	0.832
Crohn’s disease	96 (76.8)	513 (75.4)		
Crohn’s disease, location				
Small intestine	13 (10.4)	63 (9.3)	0.01 (0.00; 0.09)	0.816
Large intestine	11 (8.8)	37 (5.4)	0.05 (0.00; 0.14)	0.211
Ileocecal	79 (63.2)	431 (63.4)	0.00 (0.00; 0.08)	>0.999
Ulcerative colitis, location				
Pancolitis	25 (20.0)	158 (23.2)	0.03 (0.00; 0.09)	0.498
Left-sided	4 (3.2)	9 (1.3)	0.05 (0.00; 0.15)	0.128
Proctitis	3 (2.4)	19 (2.8)	0.01 (0.00; 0.07)	>0.999
Steroids				
Dependent	108 (86.4)	632 (92.9)	0.09 (0.01; 0.17)	**0.022**
Resistant	17 (13.6)	48 (7.1)		
5-ASA	35 (28.0)	209 (30.7)	0.02 (0.00; 0.09)	0.613
Steroids	68 (54.4)	431 (63,3)	0.07 (0.01; 0.14)	0.072
Thiopurine	113 (90.4)	575 (84.6)	0.06 (0.01; 0.12)	0.118
Regular dermatological visits	18 (14.4)	74 (10.9)	0.04 (0.00; 0.12)	0.326

5-ASA—5-aminosalicylic acid, SD—standard deviation, IBD—inflammatory bowel disease, IQR—interquartile range, MD—mean or median difference (patients with dermal lesions vs. patients without dermal lesions), and CI—confidence interval. Groups compared with Chi-square, Pearson’s test, or Fisher’s exact test (nominal variables) alongside Student’s *t*-test for independent groups or Mann–Whitney U test (numeric variables).

**Table 3:** Logistic regression outcomes for any dermal lesions.

Variable	Multivariable Logistic Regression				
	Coefficient	SE	OR	95% CI	*p*
BMI, kg/m^2^	0.07	0.03	1.08	1.02–1.14	**0.007**
Crohn’s disease, location					
Large intestine	0.68	0.36	1.97	0.92–3.90	0.063
Ulcerative colitis, location					
Left-sided	1.11	0.62	3.04	0.80–9.74	0.072
Thiopurine	0.56	0.33	1.74	0.95–3.47	0.089
Steroids, dependent (vs. resistant)	−0.81	0.31	0.45	0.25–0.83	**0.008**

BMI—body mass index, CI—confidence interval, OR—odds ratio, and SE—standard error.

## Data Availability

The data are unavailable due to privacy or ethical restrictions.

## References

[1] Łodyga M., Eder P., Gawron-Kiszka M., Dobrowolska A., Gonciarz M., Hartleb M., Kłopocka M., Małecka-Wojciesko E., Radwan P., Reguła J. (2021). Guidelines for the management of patients with Crohn’s disease. Recommendations of the Polish Society of Gastroenterology and the Polish National Consultant in Gastroenterology. Prz. Gastroenterol..

[2] Eder P., Łodyga M., Gawron-Kiszka M., Dobrowolska A., Gonciarz M., Hartleb M., Kłopocka M., Małecka-Wojciesko E., Radwan P., Reguła J. (2023). Guidelines for the management of ulcerative colitis. Recommendations of the Polish Society of Gastroenterology and the Polish National Consultant in Gastroenterology. Prz. Gastroenterol..

[3] Raine T., Bonovas S., Burisch J., Kucharzik T., Adamina M., Annese V., Bachmann O., Bettenworth D., Chaparro M., Czuber-Dochan W. (2022). ECCO Guidelines on Therapeutics in Ulcerative Colitis: Medical Treatment. J. Crohn’s Colitis.

[4] Torres J., Bonovas S., Doherty G., Kucharzik T., Gisbert J.P., Raine T., Adamina M., Armuzzi A., Bachmann O., Bager P. (2020). ECCO Guidelines on Therapeutics in Crohn’s Disease: Medical Treatment. J. Crohn’s Colitis.

[5] Gordon H., Biancone L., Fiorino G., Katsanos K.H., Kopylov U., Al Sulais E., Axelrad J.E., Balendran K., Burisch J., de Ridder L. (2023). ECCO Guidelines on Inflammatory Bowel Disease and Malignancies. J. Crohn’s Colitis.

[6] Bousis D., Verras G.I., Bouchagier K., Antzoulas A., Panagiotopoulos I., Katinioti A., Kehagias D., Kaplanis C., Kotis K., Anagnostopoulos C.N. (2023). The role of deep learning in diagnosing colorectal cancer. Prz. Gastroenterol..

[7] Chlorogiannis D.D., Verras G.I., Tzelepi V., Chlorogiannis A., Apostolos A., Kotis K., Anagnostopoulos C.N., Antzoulas A., Davakis S., Vailas M. (2023). Tissue classification and diagnosis of colorectal cancer histopathology images using deep learning algorithms. Is the time ripe for clinical practice implementation?. Prz. Gastroenterol..

[8] Lewandowski K., Kaniewska M., Więcek M., Panufnik P., Tulewicz-Marti E., Głuszek-Osuch M., Ciechanowicz P., Walecka I., Rydzewska G. (2024). Dermal lesions associated with anti-tumor necrosis factor-α therapy in patients with inflammatory bowel disease: Findings from an inflammatory bowel disease tertiary center in Poland. Pol. Arch. Intern. Med..

[9] Cushing K.C., Du X., Chen Y., Stetson L.C., Kuppa A., Chen V.L., Kahlenberg J.M., Gudjonsson J.E., Vanderwerff B., Higgins P.D.R. (2022). Inflammatory Bowel Disease Risk Variants Are Associated with an Increased Risk of Skin Cancer. Inflamm. Bowel Dis..

[10] Kucharzik T., Ellul P., Greuter T., Rahier J.F., Verstockt B., Abreu C., Albuquerque A., Allocca M., Esteve M., Farraye F.A. (2021). ECCO Guidelines on the Prevention, Diagnosis, and Management of Infections in Inflammatory Bowel Disease. J. Crohn’s Colitis.

[11] Sebastian S., Neilaj S. (2019). Practical guidance for the management of inflammatory bowel disease in patients with cancer. Which treatment?. Ther. Adv. Gastroenterol..

[12] Russomanno K., Abdel Azim S., Patel V.A. (2023). Immunomodulators for Non-Melanoma Skin Cancers: Updated Perspectives. Clin. Cosmet. Investig. Dermatol..

[13] Farraye F.A., Melmed G.Y., Lichtenstein G.R., Kane S.V. (2017). ACG Clinical Guideline: Preventive Care in Inflammatory Bowel Disease. Am. J. Gastroenterol..

[14] Johnson M.M., Leachman S.A., Aspinwall L.G., Cranmer L.D., Curiel-Lewandrowski C., Sondak V.K., Stemwedel C.E., Swetter S.M., Vetto J., Bowles T. (2017). Skin cancer screening: Recommendations for data-driven screening guidelines and a review of the US Preventive Services Task Force controversy. Melanoma Manag..

[15] Collins L., Asfour L., Stephany M., Lear J.T., Stasko T. (2019). Management of Non-melanoma Skin Cancer in Transplant Recipients. Clin. Oncol..

[16] Lam K., Coomes E.A., Nantel-Battista M., Kitchen J., Chan A.W. (2019). Skin cancer screening after solid organ transplantation: Survey of practices in Canada. Am. J. Transplant..

[17] Huang S.Z., Liu Z.C., Liao W.X., Wei J.X., Huang X.W., Yang C., Xia Y.H., Li L., Ye C., Dai S.X. (2019). Risk of skin cancers in thiopurines-treated and thiopurines-untreated patients with inflammatory bowel disease: A systematic review and meta-analysis. J. Gastroenterol. Hepatol..

[18] Singh S., Nagpal S.J., Murad M.H., Yadav S., Kane S.V., Pardi D.S., Talwalkar J.A., Loftus E.V. (2014). Inflammatory bowel disease is associated with an increased risk of melanoma: A systematic review and meta-analysis. Clin. Gastroenterol. Hepatol..

[19] Singh H., Nugent Z., Demers A.A., Bernstein C.N. (2011). Increased risk of nonmelanoma skin cancers among individuals with inflammatory bowel disease. Gastroenterology.

[20] Malik T.F., Aurelio D.M. (2023). Extraintestinal Manifestations of Inflammatory Bowel Disease. StatPearls.

[21] Martins L.M.S., Perez M.M., Pereira C.A., Costa F.R.C., Dias M.S., Tostes R.C., Ramos S.G., de Zoete M.R., Ryffel B., Silva J.S. (2018). Interleukin-23 promotes intestinal T helper type17 immunity and ameliorates obesity-associated metabolic syndrome in a murine high-fat diet model. Immunology.

[22] Schäffler H., Blattmann T., Findeisen A., Meinel F.G., Meyer-Bahlburg A., Lamprecht G., Steinmüller-Magin L., Trauzeddel R., Emmert S. (2019). PAPA-Syndrom mit Morbus Crohn und primär sklerosierender Cholangitis/Autoimmunhepatitis-Overlap-Syndrom [PAPA syndrome with Crohn’s disease and primary sclerosing cholangitis/autoimmune hepatitis overlap syndrome]. Hautarzt.

[23] Weizman A.V., Sharma R., Afzal N.M., Xu W., Walsh S., Stempak J.M., Nguyen G.C., Croitoru K., Steinhart A.H., Silverberg M.S. (2018). Stricturing and Fistulizing Crohn’s Disease Is Associated with Anti-tumor Necrosis Factor-Induced Psoriasis in Patients with Inflammatory Bowel Disease. Dig. Dis. Sci..

[24] Patil S.A., Cross R.K. (2018). More Skin in the Game: Screening for Skin Cancer in IBD Patients. Dig. Dis. Sci..

[25] Siegel R.L., Miller K.D., Jemal A. (2018). Cancer statistics, 2018. CA Cancer J. Clin..

[26] Aitken J.F., Elwood M., Baade P.D., Youl P., English D. (2010). Clinical whole-body skin examination reduces the incidence of thick melanomas. Int. J. Cancer.

[27] Wernli K.J., Henrikson N.B., Morrison C.C., Nguyen M., Pocobelli G., Whitlock E.P. (2016). Screening for Skin Cancer in Adults: An Updated Systematic Evidence Review for the U.S. Preventive Services Task Force.

[28] Lemaitre M., Kirchgesner J., Rudnichi A., Carrat F., Zureik M., Carbonnel F., Dray-Spira R. (2017). Association between Use of Thiopurines or Tumor Necrosis Factor Antagonists Alone or in Combination and Risk of Lymphoma in Patients with Inflammatory Bowel Disease. JAMA.

[29] Glazer A.M., Rigel D.S. (2017). Analysis of Trends in Geographic Distribution of US Dermatology Workforce Density. JAMA Dermatol..

[30] Finnane A., Dallest K., Janda M., Soyer H.P. (2017). Teledermatology for the Diagnosis and Management of Skin Cancer: A Systematic Review. JAMA Dermatol..

[31] Hamidi R., Peng D., Cockburn M. (2010). Efficacy of skin self-examination for the early detection of melanoma. Int. J. Dermatol..

[32] Trovato E., Dragotto M., Capalbo E., Cartocci A., Rubegni P., Calabrese L. (2024). Risk of Skin Cancer in Patients with Psoriasis: Single-Center Retrospective Study Comparing Anti-TNFα and Phototherapy. J. Clin. Med..

